# Reactive arthritis and silent thyroiditis following SARS‐CoV‐2 infection: Case report and review of the literature

**DOI:** 10.1002/ccr3.5430

**Published:** 2022-02-07

**Authors:** Jacob Quaytman, Usha Gollamudi, Noah Bass, Shashank Suresh

**Affiliations:** ^1^ 6614 Division of General Internal Medicine Department of Medicine University of Pittsburgh Pittsburgh Pennsylvania USA; ^2^ Internal Medicine University of Pittsburgh Medical Center Pittsburgh Pennsylvania USA; ^3^ 6614 Division of Rheumatology and Clinical Immunology Department of Medicine University of Pittsburgh Pittsburgh Pennsylvania USA; ^4^ Community Medicine University of Pittsburgh Medical Center Pittsburgh Pennsylvania USA

**Keywords:** allergy and immunology, endocrinology and metabolic disorders, infectious diseases

## Abstract

SARS‐CoV‐2 has been implicated in the development of reactive arthritis (ReA) days to weeks following exposure or infection. We present a case of ReA with enthesitis and subsequent silent thyroiditis in a patient following infection with SARS‐CoV‐2, along with a review of the published cases of SARS‐CoV‐2‐related ReA.

## Graphical abstract

SARS‐CoV‐2 is increasingly being recognized as a cause of reactive arthritis and thyroiditis. Clinicians should be aware of these atypical presentations of COVID‐19.

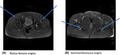

## 1 INTRODUCTION

Reactive arthritis (ReA) is a seronegative spondyloarthropathy following urogenital and gastrointestinal tract infection over several weeks, typically characterized by large joint oligoarthritis and negative synovial fluid cultures.^1^ ReA has been described following gonococcal and chlamydial urethritis as well as enteritis caused by certain *Campylobacter*, *Shigella*, and *Salmonella* species, in association with HLA‐B27 positivity in 50%–80% of patients.^2^, ^3^

Cases of ReA associated with infection with severe acute respiratory syndrome coronavirus 2 (SARS‐CoV‐2) are being described in the literature.^4^, ^5^ These cases are characterized by asymmetric oligoarthritis one to six weeks following symptomatic respiratory infection with SARS‐CoV‐2.^6^ We describe here a case of ReA with silent thyroiditis following SARS‐CoV‐2 infection.

## 2 CASE DESCRIPTION

A 48‐year‐old man unvaccinated against COVID‐19 who worked in the fracking industry with past medical history of celiac artery compression syndrome and stage IB left upper lobe lung adenocarcinoma in remission after resection had been experiencing three weeks of progressive myalgias, arthralgias, muscle stiffness and cramping, and paresthesias involving primarily his bilateral lower extremities but also his shoulders beginning in January 2021. He tried ibuprofen and topical diclofenac without relief and ultimately his symptoms prevented him from working. He had multiple exposures to coworkers with COVID‐19 prior to his symptoms but did not receive any testing himself.

He eventually was hospitalized in early February 2021. He was noted to have primarily distal lower extremity weakness greater on the left than right, bilateral absent ankle jerks, and bilateral moderately decreased vibration sense in his toes. Upper extremity strength and sensation were normal. Creatine kinase (CK) was 73 U/L, C‐reactive protein (CRP) was 3.5 mg/L, erythrocyte sedimentation rate (ESR) was 21 mm/h, and B12 was 468 pg/mL. Noncontrast magnetic resonance imaging (MRI) of thoracic and lumbar spine showed multilevel degenerative disc disease without severe canal stenosis or cord compression. Lumbar puncture (LP) revealed normal opening pressure, no nucleated cells, protein 54 mg/dl, glucose 60 mg/dl, and negative cerebrospinal fluid (CSF) gram stain and culture. Electrodiagnostic studies (EMG/NCS) and SRS‐CoV‐2 testing were not performed. Neurologist felt that the presentation was consistent with an acute inflammatory demyelinating polyradicular neuropathy and recommended intravenous immunoglobulin (IVIg), but the patient did not prefer treatment without a definitive diagnosis.

**FIGURE 1:**
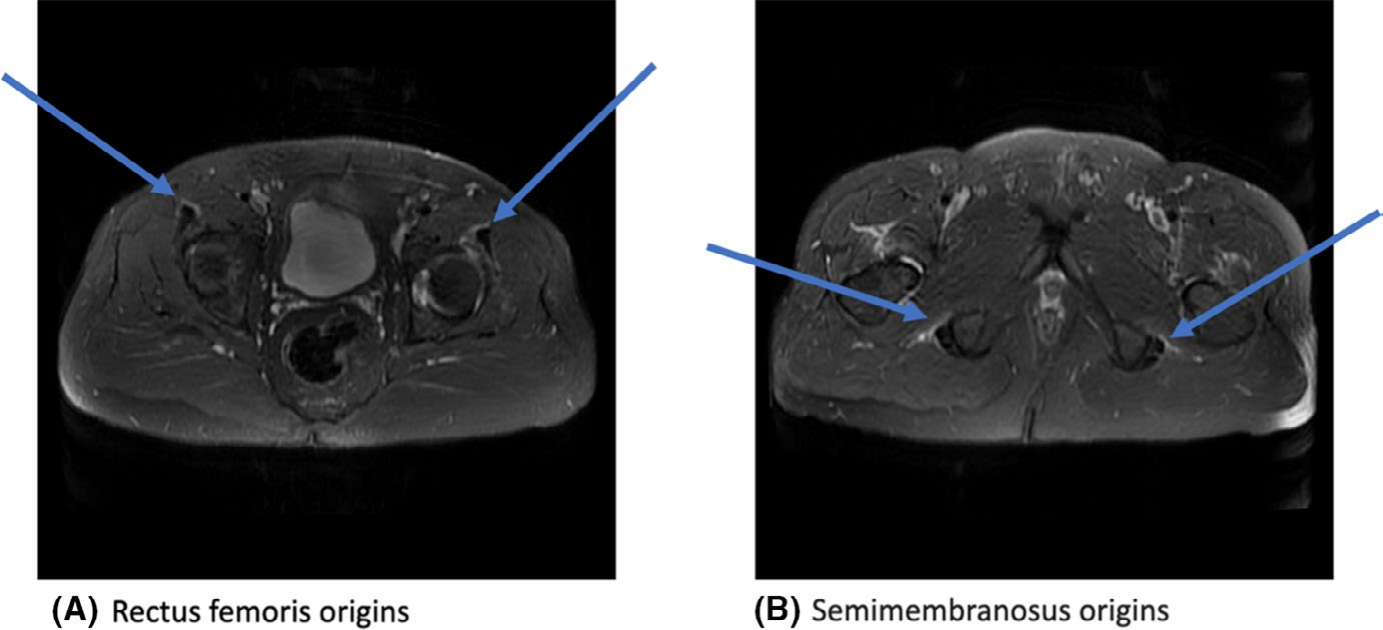
Initial MRI pelvis with and without contrast with nonspecific edema and enhancement along the bilateral semimembranosus and rectus femoris origins

He came back to the hospital three days later after again being unable to work. Muscle bulk and tone were normal, and bilateral lower extremity strength was 4/5 with limitation due to pain. Sensation was intact to light touch. He developed fever up to 39.5°C and tested positive for SARS‐CoV‐2 on PCR testing. White blood cell count was initially 4,700 cells/ul with lymphopenia to 500 cells/ul, but this later recovered. Creatinine, liver function tests, and complements were unremarkable. MRI thoracic and lumbar spine with and without contrast showed degenerative changes at L4‐L5 with mild spinal canal narrowing and moderate narrowing of the right neural foramen. MRI pelvis with and without contrast revealed edema and enhancement along the bilateral semimembranosus and rectus femoris origins and hip joint capsule (Figure 1). EMG/NCS of the right upper and lower extremities revealed no evidence of acute inflammatory demyelinating polyneuropathy, myopathy, or radiculopathy. LP was repeated showing clear fluid, 1 WBC, 0 RBCs, glucose 66 mg/dl, and protein 38 mg/dl. Serum and CSF Lyme, autoimmune, viral, and paraneoplastic panels were unremarkable. Rheumatologic workup was negative for HLA‐B27 and antibody testing was negative (ANA, dsDNA, Smith, RNP, Chromatin, SS‐A/SS‐B, ANCA, and acetylcholine receptor). Serum RPR and urine chlamydia and gonorrhea testing were negative. Ultimately, rheumatologist diagnosed the patient with reactive arthritis secondary to his SARS‐CoV‐2 infection. It was thought that the patient had initially been infected in January after his work exposures and his SARS‐CoV‐2 PCR test remained positive because he was within 90 days of his initial infection. He was discharged on nonsteroidal anti‐inflammatory drugs (NSAIDs) and prednisone 15 mg twice daily with significant relief in pain but not complete return to baseline functioning.

**FIGURE 2:**
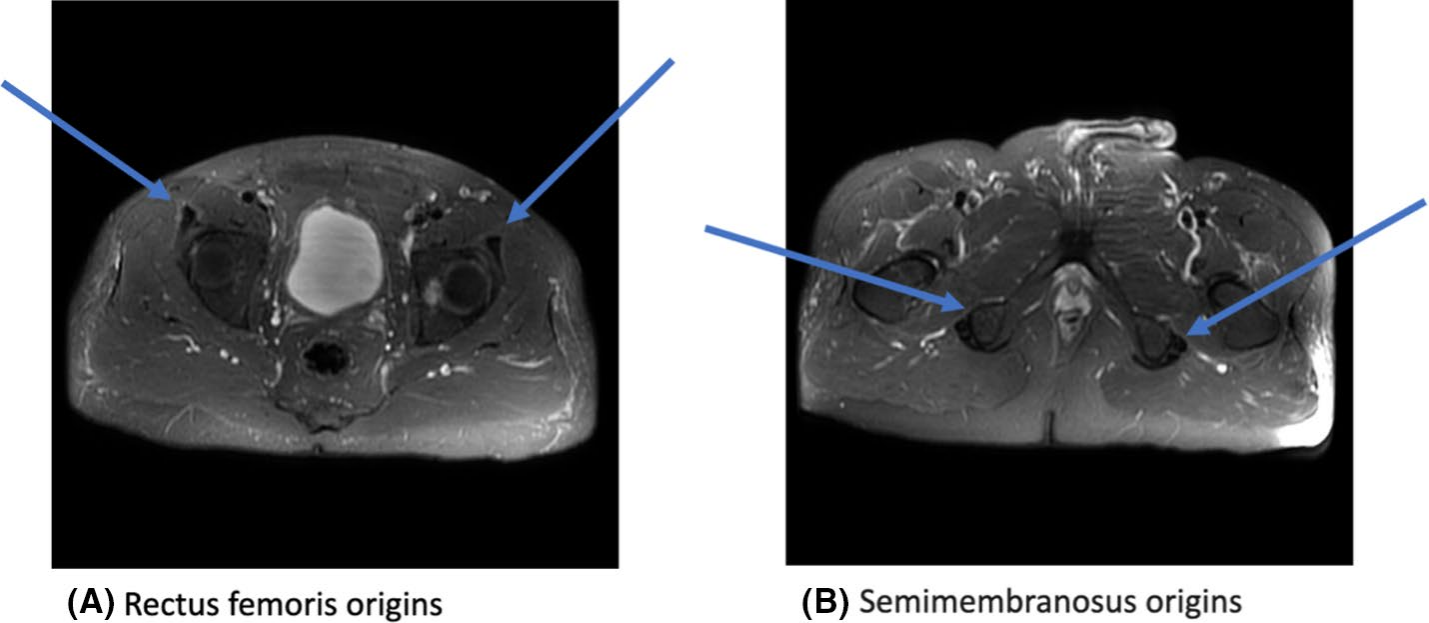
MRI pelvis with and without contrast one month after the initial MRI and a course of steroids showing complete resolution of the bilateral semimembranosus and left rectus femoris nonspecific edema and enhancement along with minimal residual right rectus femoris nonspecific edema and enhancement

Over the next few months, the patient's bilateral hip and shoulder pain returned as his steroids were tapered. Follow‐up MRI pelvis one month after his initial MRI showed the resolution of the enthesitis (Figure 2). A diagnosis of polymyalgia rheumatica or giant cell arteritis was considered but the patient declined a temporal artery biopsy. He was trialed on sulfasalazine without success and ultimately was maintained on prednisone and minocycline, with partial resolution of his symptoms.

**FIGURE 3:**
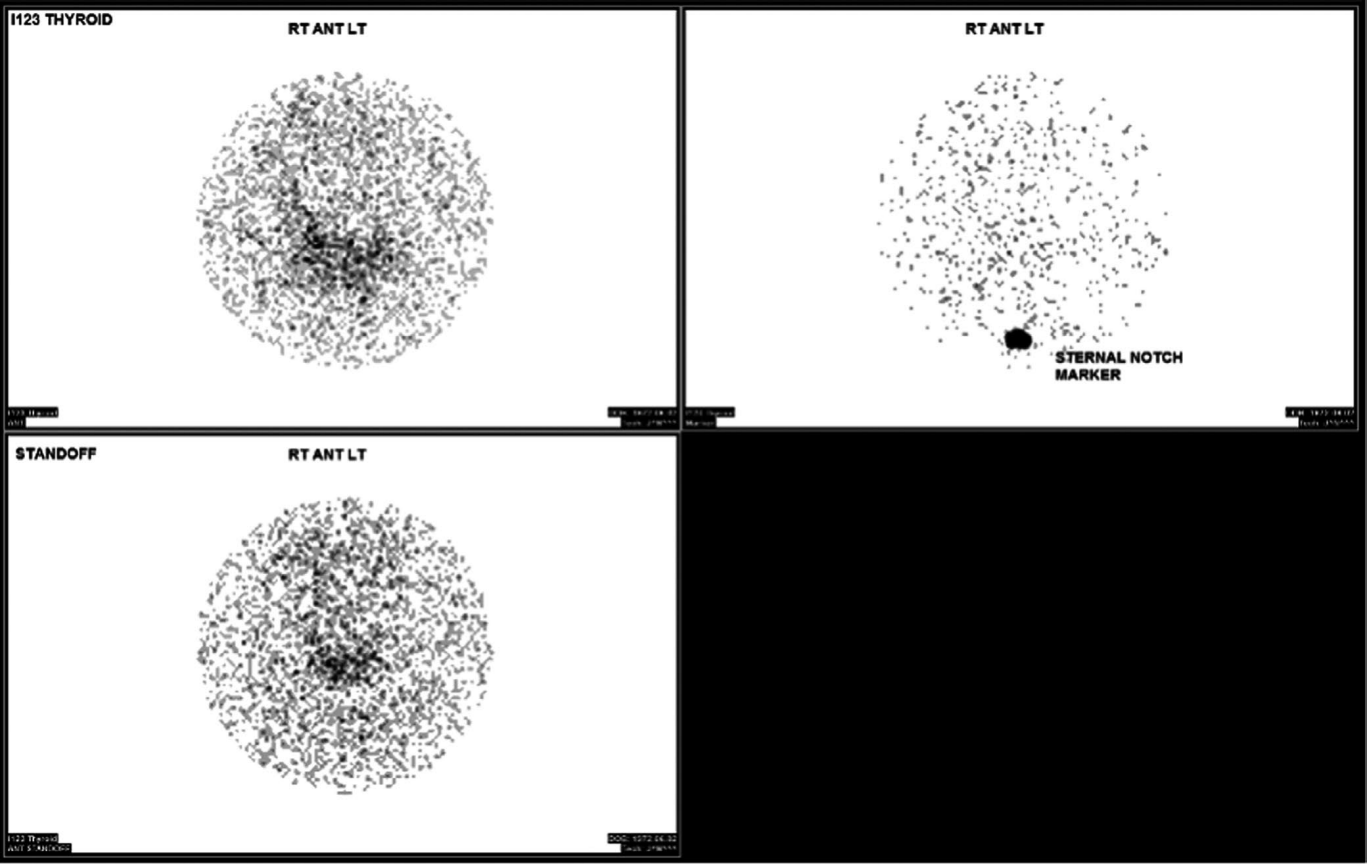
Radioactive iodine uptake scan showing diffusely reduced uptake at 4 and 24 h consistent with thyroiditis

During hospitalization for increased pain in April 2021, he was also noted to have some heat intolerance, palpitations, and increased anxiety. TSH was 0.015 and free T4 was elevated to 1.92 and at endocrinology follow‐up in May 2021, TSH was suppressed to <0.005 and free T4 was up to 1.95. He had no anterior neck pain or swelling. Chest CT angiography done in February 2021 as part of his lung cancer follow‐up did not note any thyroid nodules. Since thyroid peroxidase (TPO), thyroglobulin (Tg) antibodies, and thyroid‐stimulating immunoglobulin (TSI) were negative, autoimmune thyroid disease was less likely. Radioactive iodine uptake scan showed diffusely decreased uptake at 4 and 24 h (Figure 3), consistent with silent thyroiditis triggered by SARS‐CoV‐2 infection. He received supportive treatment with propranolol and by November 2021; the patient was euthyroid.

## 3 DISCUSSION

We present a case of reactive arthritis and silent thyroiditis that developed after SARS‐CoV‐2 infection. The patient had elevated inflammatory markers and MRI evidence of synovitis and enthesitis that resolved after glucocorticoids. He developed a transient period of hyperthyroidism that ultimately resolved with supportive care. Symptoms recurred with tapering the prednisone, and sulfasalazine was initiated before transitioning to minocycline and maintenance steroids.

The patient developed muscle pain and weakness initially in early January 2021, about three weeks before his first SARS‐CoV‐2 PCR test was performed. He had multiple coworkers who were diagnosed with COVID‐19 in January and was likely initially infected at this time, but the patient did not seek out testing because of his atypical presentation. His positive SARS‐CoV‐2 PCR test a few weeks into his illness likely reflected an earlier infection, as it is known that PCR tests can remain positive for up to 90 days after the initial infection and not necessarily reflect acute infection. The patient did not have the respiratory symptoms of typical COVID‐19, but we attributed his reactive arthritis and silent thyroiditis to SARS‐CoV‐2 after extensive serologic and imaging workup for other etiologies was unrevealing.

To date, there have been at least 16 cases reported of ReA associated with SARS‐CoV‐2 infection. A PubMed database search was conducted on April 21, 2021, with the search term “COVID‐19 reactive arthritis”, and case reports and reviews of oligoarthritis in adult patients following SARS‐CoV‐2 infection were selected for further review. Additional reports that are not in the database search but referenced in the selected publications were also included. The articles are summarized in Table 1.

**TABLE 1:** Reported cases and series of reactive arthritis following SARS‐CoV‐2 infection in PubMed database

Author	Year	Timeline of arthritis after SARS‐CoV‐2 positivity	Treatment	Outcome
Ono et al.^6^	2020	Ankle arthritis and Achilles tendon enthesitis 3 weeks after positive test	NSAIDs and intra‐articular steroids	Clinical improvement
Saricaoglu et al.^7^	2020	Arthritis of MTP and interphalangeal joints of the feet 1 week after positive test	NSAIDs	Resolution
Jali^8^	2020	Arthritis of interphalangeal joints of the hands 3 weeks after positive test	Celecoxib	Resolution
Liew et al.^9^	2020	Arthritis of right knee at time of positive test, exposure thought to be one week prior	Etoricoxib and intra‐articular steroids	Clinical improvement
Fragata and Mourao^4^	2020	Arthritis of MCP and interphalangeal joints of the hands 4 weeks after first positive test	Steroids	Resolution
Honge et al.^5^	2021	Fever and knee, ankle arthritis 2 weeks after positive test	Ibuprofen and prednisolone	Resolution
Coath et al ^10^	2021	Sacroiliac arthritis at time of positive test, with preceding exposure and suggestive symptoms of SARS‐CoV−2	Intramuscular methylprednisolone and diclofenac	Resolution
Schenker et al.^11^	2021	Arthritis of ankles, wrists, and knees 2–3 weeks following positive test	Prednisolone	Resolution
De Stefano et al.^12^	2020	Elbow arthritis and psoriasis‐form rash 1–2 weeks following positive test	NSAIDs and topical steroids for rash	Resolution
Danssaert et al.^13^	2020	Hand arthritis 1–2 weeks following positive test	Topical diclofenac, hydromorphone, gabapentin, and later tramadol and occupational therapy	Clinical improvement
Gasparotto et al.^14^	2021	Ankle, knee, and hip arthritis 4–5 weeks after positive test	Ibuprofen	Resolution
Parisi et al.^15^	2020	Ankle arthritis 3–4 weeks following positive test	Ibuprofen	Clinical improvement
Yokogawa et al.^16^	2020	Fever and wrist, shoulder, and knee arthritis 2 weeks after positive test	None	Spontaneous resolution
Lopez‐Gonzalez et al.^17^	2020	Four cases occurring after symptom onset First MTP (8 days) Ankle (19 days) Knees (8 days) Knee and ankle (27 days)	Colchicine and intra‐articular steroids Colchicine and oral prednisone Intra‐articular steroids Colchicine	Resolution in all cases
Di Carlo et al.^18^	2021	Ankle arthritis 5 weeks after positive test	Methylprednisolone	Clinical improvement (continued on steroids as of publication)
Sureja and Nandamuri^19^	2021	Hand, knee, ankle, and foot arthritis 2 weeks after positive test	NSAIDs, tapering steroids, opiates	Resolution

Clinical manifestations of ReA are variable and include several articular as well as extra‐articular manifestations, including asymmetric mono‐ or oligo‐arthritis (involving 2–4 joints) days to weeks after enteric or genitourinary pathogens, without crystalline arthritis.^3^, ^20^, ^21^ ReA is typically an oligoarthritis of lower extremity joints (as in our case) but may present with polyarthritis of hand joints with dactylitis.^20^ The axial skeleton may be involved with sacroiliac joint synovitis and enthesitis, and extra‐articular involvement includes uveitis/conjunctivitis, urethritis/cervicitis, rash, aphthous ulcers, and more rarely aortitis and cardiac conduction abnormalities.^20^

Both silent and subacute thyroiditis after SARS‐CoV‐2 infection have been reported. Subacute thyroiditis often presents with fever and anterior neck pain whereas silent thyroiditis is painless, but both are self‐limited.^22^, ^23^, ^24^ Our patient was febrile initially but had no neck pain and normal thyroid function initially. He went on to develop complete TSH suppression with an elevated free T4 without any associated neck pain, most consistent with a silent thyroiditis. Steroid use could have contributed to low TSH but not elevated free T4. The Jod–Basedow effect from iodinated contrast could not explain persistent hyperthyroidism three months after exposure.

Diagnosis of thyroiditis is made clinically with laboratory investigations, and in some cases, a radioactive iodine uptakes scan to rule out Graves’ disease.^24^ Histopathology in SARS‐CoV‐2 thyroiditis shows interstitial infiltration by lymphocytes and destruction of follicular cells.^24^, ^25^ The treatment of subacute and silent thyroiditis is supportive as the thyroid dysfunction in most cases is transient. NSAIDs can be used in mild cases of neck pain and glucocorticoids for severe cases, while beta blockers and levothyroxine can be used for tachyarrhythmias and hypothyroidism.^24^

Treatment of ReA is aimed at reducing articular and extra‐articular inflammation and increasing mobility: NSAIDs are the first‐line therapy, with systemic corticosteroids reserved for refractory or severe disease of long duration.^26^ Methotrexate, sulfasalazine, and azathioprine also have demonstrated efficacy in treating disease refractory to corticosteroids.^26^ Our patient had significant relief on an initial course of NSAIDs required several prednisone tapers due to the severity and duration of his disease (overall course greater than 3 months). He was initiated on sulfasalazine without success and was later started on minocycline. As of his most recent follow‐up, he had not completely returned to baseline.

## 4 CONCLUSION

ReA typically manifests as oligoarthritis of the lower extremities several weeks following enteric or genitourinary infection, and in some cases, viral and streptococcal infections. Our patient had asymmetric oligoarthritis and imaging showing enthesitis with elevated acute phase reactants after SARS‐CoV‐2 infection, along with suppressed TSH and elevated free T4, all of which favors the diagnosis of ReA and silent thyroiditis secondary to SARS‐CoV‐2 infection. He continues to have incomplete clinical resolution of the arthralgias despite resolved enthesitis. Several cases of ReA and thyroiditis due to the pandemic virus SARS‐CoV‐2 have been published, and it is expected that more cases and series will be reported in the coming months given the widespread global scale of the pandemic.

## CONFLICT OF INTEREST

There are no conflicts of interest to report.

## AUTHOR CONTRIBUTIONS

Jacob Quaytman and Shashank Suresh gathered data and prepared manuscript. Usha Gollamudi and Noah Bass reviewed and edited the manuscript.

## ETHICAL APPROVAL

The research complies with the Declaration of Helsinki.

## CONSENT

Written consent was obtained from the patient described in this case report prior to publication.

## Data Availability

All data are included in this article but additional data available on request due to privacy/ethical restrictions.
